# Symptom-Based Ebola Risk Score for Ebola Virus Disease, Conakry, Guinea

**DOI:** 10.3201/eid2406.171812

**Published:** 2018-06

**Authors:** Brecht Ingelbeen, Anja De Weggheleire, Michel Van Herp, Johan van Griensven

**Affiliations:** Médecins sans Frontières, Conakry, Guinea (B. Ingelbeen, M. Van Herp);; Institute of Tropical Medicine, Antwerp, Belgium (A. De Weggheleire, J. van Griensven);; European Programme for Intervention Epidemiology Training (EPIET), European Centre for Disease Prevention and Control, Stockholm, Sweden (B. Ingelbeen).

**Keywords:** Ebola virus, Ebola virus disease, hemorrhagic fever, Ebola, risk, viruses

**To the Editor:** In their article, Oza et al. proposed a score to risk-stratify Ebola virus disease (EVD) suspected cases while patients in an Ebola treatment center await laboratory confirmation (*1*). The Ebola symptom-based risk (ESR) score, consisting of 6 symptoms (conjunctivitis, diarrhea, nausea/vomiting, headache, difficulty breathing, loss of appetite), performed well in internal validation, but no external validation was done.

 We evaluated the proposed ESR score on 805 EVD-positive and 1,506 EVD-negative case-patients in the Conakry Ebola Treatment Center (ETC), Conakry, Guinea (*2*). The ESR score yielded an area under the curve of 0.58 (95% CI 0.56–0.61), which is lower than the 0.83 (95% CI 0.79–0.86) Oza et al. reported (Technical Appendix Figure). Using the proposed risk thresholds (i.e., low risk if score <0, medium risk if score = 0, and high risk if score >0), 371 (46%) EVD-positive patients of the Conakry ETC were classified as high risk and 647 (43%) EVD-negative patients as low risk. However, negative and positive predictive values were generally low (Technical Appendix Table). Reasons for poor validation could include differences in applying the general EVD suspect case definition (integration of patients’ contact history); in patient characteristics because organization and access to care for EVD and non-EVD illness was different (patients in holding centers or ETC); in the quality of data collection (symptoms are entirely self-reported); and in underlying diseases of EVD-negative patients. 

Our findings underline the importance of external validation in various settings before risk scores are applied outside of the setting within which they were developed, as well as the need to incorporate patient contact history into predictive models. Point-of-care EVD diagnostic platforms can perform reliable confirmatory testing within 90 minutes (*3*). We argue that, by integrating rapid confirmatory testing in triage, providers can avoid classifying patients by their likelihood of infection with Ebola virus while waiting for laboratory confirmation. 

Dr. Ingelbeen, a pharmacist and epidemiologist, worked during the 2014–2016 Ebola outbreak with Médecins sans Frontières in Guinea. He is a European Programme for Intervention Epidemiology Training (EPIET) fellow at Santé publique France. His research interests include emerging and vectorborne infectious diseases.

Technical Appendix Additional information about validation testing for Ebola symptom-based risk scores, Guinea.
