# Supplementary material for: Symptom-Based Ebola Risk Score for Ebola Virus Disease, Conakry, Guinea
Source: Emerg Infect Dis. 2018 Jun;24(6):1163. doi: 10.3201/eid2406.171812 (PMC6004844; doi:10.3201/eid2406.171812)
Supplement: Technical Appendix — Additional information about validation testing for Ebola symptom-based risk scores, Guinea. [file 17-1812-Techapp-s1.pdf]

Article DOI: https://doi.org/10.3201/eid2406.171812

Symptom-Based Ebola Risk Score for Ebola Virus Disease, Conakry, Guinea

Technical Appendix

Technical Appendix Table. Characteristics of Ebola symptom-based risk scores to predict Ebola virus disease confirmation among patients admitted to the Conakry Ebola Treatment Center, Guinea, 2014–2015*

ESR score	% EVD negative	% EVD positive	Sensitivity (95% CI)	Specificity (95% CI)	PPV (95% CI)	NPV (95% CI)
>-3	1	0	100 (100–100)	0 (0–0)	NA	NA
>-2	13	9	100 (100–100)	1 (0–1)	35 (33–37)	100 (74–100)
>-1	29	24	91 (89–93)	14 (12–16)	36 (34–38)	75 (70–80)
>0	23	21	67 (64–71)	43 (40–46)	39 (36–41)	71 (68–74)
>1	17	19	46 (43–50)	66 (64–69)	42 (39–46)	70 (67–72)
>2	11	15	27 (24–30)	83 (81–85)	46 (42–51)	68 (66–70)
>3	4	8	12 (10–14)	95 (94–96)	55 (47–62)	67 (65–69)
>4	1	3	3 (2–5)	99 (99–99)	61 (46–76)	66 (64–68)
>5	0	0	0 (0–1)	100 (99–100)	38 (9–76)	65 (63–67)

*ESR, Symptom-Based Ebola Risk; EVD, Ebola virus disease; PPV, positive predictive value; NPV, negative predictive value; NA, not available

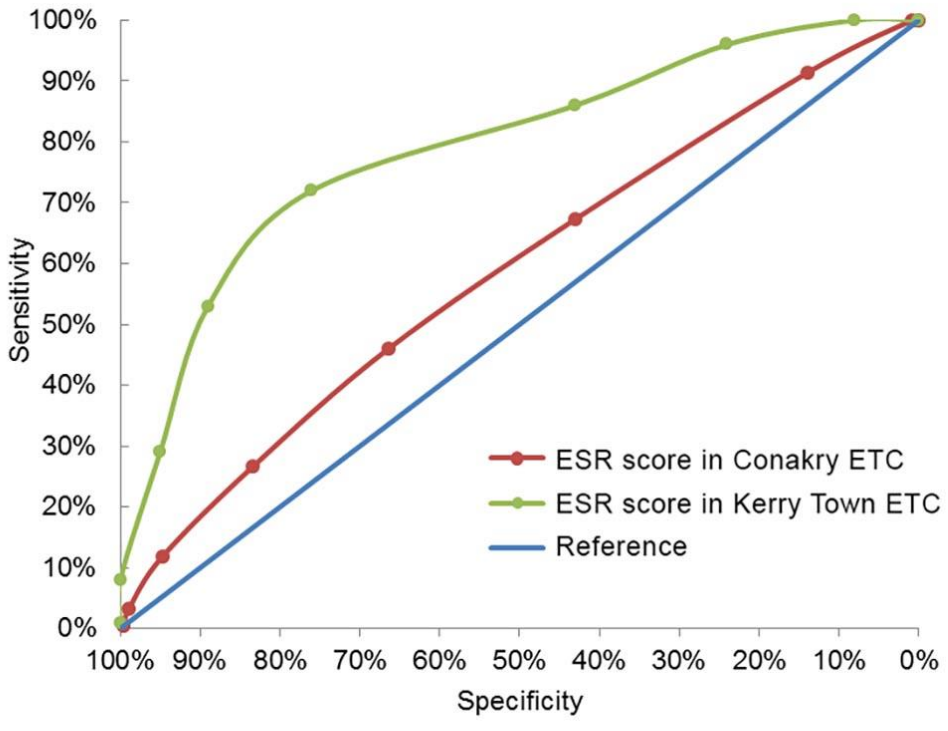

Technical Appendix Figure. Receiver operating characteristic curves to identify risk for confirmed Ebola virus disease among patients admitted to the Conakry treatment center, Guinea, and to the Kerry Town treatment center, Sierra Leone, 2014–15.ETC, Ebola treatment center. The diagonal reference line indicates success expected on the basis of chance (AUC = 50%).
